# What information is provided in transcripts and Medical Student Performance Records from Canadian Medical Schools? A retrospective cohort study

**DOI:** 10.3402/meo.v19.25181

**Published:** 2014-09-08

**Authors:** Jason A. Robins, Matthew D. F. McInnes, Kaisra Esmail

**Affiliations:** 1 The Ottawa Hospital Research Institute, Ottawa, Ontario, Canada; 2 Department of Radiology, University of Ottawa, Ottawa, Ontario, Canada

**Keywords:** transcript, dean's letter, Medical Student Performance Record, evaluation, residency

## Abstract

**Background:**

Resident selection committees must rely on information provided by medical schools in order to evaluate candidates. However, this information varies between institutions, limiting its value in comparing individuals and fairly assessing their quality. This study investigates what is included in candidates’ documentation, the heterogeneity therein, as well as its objective data.

**Methods:**

Samples of recent transcripts and Medical Student Performance Records were anonymised prior to evaluation. Data were then extracted by two independent reviewers blinded to the submitting university, assessing for the presence of pre-selected criteria; disagreement was resolved through consensus. The data were subsequently analysed in multiple subgroups.

**Results:**

Inter-rater agreement equalled 92%. Inclusion of important criteria varied by school, ranging from 22.2% inclusion to 70.4%; the mean equalled 47.4%. The frequency of specific criteria was highly variable as well. Only 17.7% of schools provided any basis for comparison of academic performance; the majority detailed only status regarding pass or fail, without any further qualification.

**Conclusions:**

Considerable heterogeneity exists in the information provided in official medical school documentation, as well as markedly little objective data. Standardization may be necessary in order to facilitate fair comparison of graduates from different institutions. Implementation of objective data may allow more effective intra- and inter-scholastic comparison.

Each fall, Canadian medical schools produce Medical Student Performance Records (MSPRs) and transcripts in support of their graduates’ residency applications. Residency program selection committees throughout the country rely on this information in order to evaluate candidates. However, the information provided by each institution can vary, which may limit its value in comparing individuals and fairly assessing their quality.

Residency program directors favour objective data to guide selection strategies (1, 2). However, many medical schools across North America are moving to a pass–fail system without any objective data regarding student performance (3).

The objective of this study was to evaluate the variability of information provided to resident selection committees from each Canadian medical school in the form of medical school transcripts, performance records, and dean's letters. A secondary objective was to evaluate the proportion of schools that provide objective data regarding student performance; this was defined as letter grade, percentage grade, class average, distribution of marks, percentile, and/or class rank.

## Methods

Approval was waived by the institutional Research Ethics Board (REB). We retrospectively collected sample MSPRs and transcripts from all 17 Canadian medical schools. These documents were taken from applications to our program in the 2012 or 2013 Canadian Resident Matching Service (CaRMS) R-1 Main Residency Match, with one application selected randomly from each university, totalling 17 document packages each consisting of one MSPR and transcript; 2 years were necessary in order to obtain a complete data set. Only one application was chosen per school in order to most accurately reflect what any given program may receive (since programs may have only one applicant from a particular school), as opposed to a representative sample of all documents from that school. In order to blind the data extractors to the institution and ensure anonymity, a research assistant anonymised all files with respect to the submitting institution (they were assigned a study identification number) and personal identifying data.

A list of criteria to extract from the documents was generated by the team of investigators (XX first year resident, YY third year resident, ZZ residency program director with 6 years of post-graduate medical education experience). The criteria chosen were based on information that would be potentially useful to resident selection committees and was primarily guided by three important publications in the area of resident selection including a consensus statement from the 2010 Canadian Conference on Medical Education regarding selection for specialty training (4–6). In addition, a recent comprehensive systematic review on resident selection strategies was used as a guide regarding which additional criteria including which objective data should be included (7).

Data extracted from each document included: time, curriculum, evaluation, research, extracurricular activities, narrative, standardized examinations, and disclosure sheet (see Table 1 for a complete list of criteria). Two readers (XX and YY) independently extracted the data assessing for the presence or absence of criteria; extractors were blinded to the submitting university/medical school. Discrepancies were resolved through discussion with a third reviewer (ZZ). Inter-rater agreement was calculated.

**Table 1:** Frequency of criteria inclusion

Criteria	Frequency (%)
Time	
Date enrolled	88.24
Date of expected completion/graduation	58.82
Leave of absence/delays	52.94
Reason for absence/delays	29.41
Courses	
Compulsory courses/rotations detailed	100.00
Elective rotations detailed	70.59
Incomplete/failed courses/rotations	82.35
Original performance of failed course included	29.41
Remediation necessary	52.94
Remediation completed	41.18
Evaluation	
Type of marking scheme detailed	88.24
Letter grade	17.65
Percentage	0.00
Likert/rating scale	47.06
Pass/fail	64.71
Pass/fail/honours	23.53
Basis for comparison with others	17.65
Class average (letter grade)	11.76
Class rank (relative standing in performance, 1st through last)	0.00
Percentile (value on a scale of 100 indicating the percent of a distribution that is equal to or below it)	0.00
Distribution of marks	17.65
Individual course/rotation marks (includes electives)	100.00
Supervisor comments	88.24
Notice concerning editing	47.06
Verbatim	23.53
Edited	23.53
Academic awards	52.94
Reason for awards	29.41
Research	
Mandatory/elective detailed	35.29
Research awards	29.41
Reason for awards	0.00
Extracurricular activities	
Athletics	23.53
Arts	17.65
Student initiatives/government	52.94
Other	47.06
Narrative	
Comment by dean/other official	0.00
Professionalism statement	41.18
Mentorship statement	5.88
Standardized examinations	
MCAT	0.00
USMLE 1	0.00
Disclosure sheet	41.18

The resulting data set was analysed according to the proportion of criteria included by each school, overall frequency of inclusion of particular criteria, as well as the presence or absence of objective data, as defined above. Data analysis was done in Microsoft Excel 2010 (Microsoft Corporation; Redmond, Washington).

## Results

**Fig. 1:**
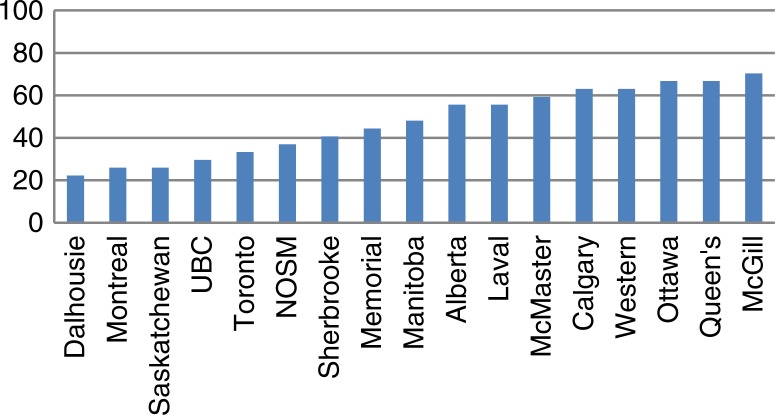
Percent adherence to selected criteria, by university (UBC=University of British Columbia; NOSM=Northern Ontario School of Medicine).

Transcripts and MSPRs from all 17 Canadian medical schools were collected. Inter-rater agreement was 92% prior to consensus discussion. None of the disagreements required discussion with the third reviewer. Overall inclusion of all of the criteria varied by school, ranging from 22.2% (Dalhousie) to 70.4% (McGill); the mean was 47.4% and the median 48.1% (Fig. 1). The frequency of inclusion of any individual criterion was highly variable (see Table 1). All schools detailed the courses that were compulsory, as well as the individual performance in each of these; none included explanations for research awards, comments by the dean (or other senior official), nor standardized examination scores.

Objective data were lacking beyond just standardized examination scores; only 17.7% of schools provided any basis for comparison of academic performance (i.e., class average, distribution of marks within class). L'Université de Montréal and L'Université de Sherbrooke included both of these details; McGill University reported the latter. None of the universities provided a class rank or percentile. The majority only provided status regarding pass or fail, without any further qualification. Fifty-three percent reported academic awards that the candidate had received while at their institution; however, only just over half of these explained the reason or performance level for which they were awarded.

In terms of curriculum, though all schools listed their mandatory rotations, only 70.6% detailed the electives undertaken by the candidate. Eighty-two percent of the universities made note of incomplete or failed courses, but only 29.4% provided details regarding original performance and remediation.

Disclosure sheets (documents which detail exactly what is included in the candidate's evaluation and where this information can be found) are not mandatory in order to complete one's CaRMS application; as a result, they were provided by only 41.2% of universities.

## Discussion

There is considerable variability regarding information provided by official medical school documentation, as well as markedly little objective data. This is highlighted by the inclusion of any form of objective data (as defined within the Introduction) by only three of the 17 institutions. The lack of objective information provided by Canadian medical schools is an interesting juxtaposition to their own admission requirements, which rely on objective data such as Medical College Admissions Test (MCAT) scores and undergraduate marks. Studies in both Canada and the US have documented that program directors prefer objective data for resident selection and few put any value on the dean's letter (1, 2). The lack of objective data from medical schools has led some programs to look for alternate sources of such data. For example, 5/16 Diagnostic Radiology residency programs in Canada require an undergraduate transcript (from a degree prior to the candidate entering medical school), and another requests one as an option as part of their residency application. This may over-emphasize undergraduate achievement at the expense of those who excelled in medical school but had no objective data to show for it.

Without objective data, selection committees are often left to rely on interviews; however, at least in the traditional sense (single session with one interviewer or panel), these have been poor predictors of candidate quality (8). This is further confirmed by the consensus statement on residency training selection arising from the Ottawa 2010 Conference which states that interviews ‘have not been shown to be robust selection measures’ (5). Though the emerging trend towards multiple mini-interviews has shown promise (4), the data remain limited. This only further underscores the need for objective data, in which MSPRs/transcripts would ideally play an important role.

An in depth literature search revealed that no comparable work has been previously performed regarding the Canadian system. However, the topic of MSPR content has been explored within the United States. In 1989, the Association of American Medical Colleges (AAMC) first published guidelines regarding the dean's letter, highlighting that this ‘is not a letter of recommendation; it is a letter of evaluation’ (9). An evaluation of their adoption performed 10 years later found that over a third of U.S. medical schools produced letters that were inadequate; the authors noted that comparative performance data were most often lacking (10). Aware of these issues, in 2002 the AAMC proposed ‘A Guide to the Preparation of the Medical Student Performance Evaluation’, in a further effort to promote consistency across universities. A repeat survey 3 years later found that although there had been improvements in adherence, ‘a sizable minority of writers are still using the MSPE as a recommendation, and too few are providing helpful comparative data’ (11). The authors posited that schools were reluctant to directly compare lower performing students with their classmates for fear of compromising their success in the Match. A separate analysis supported this hypothesis, finding that negative information available in the official transcript was withheld from the dean's letter up to one third of the time (12).

Several limitations were identified during the completion of this study. First, although the data extractors were blinded to the institution, the three francophone schools could readily be identified since the transcripts were in French. Second, the source providing the information was not always clearly defined. For example, one MSPR detailed extensive extracurricular activities, only to later explain that these were provided by the candidate themselves rather than confirmed by a school official. Additionally, further details may have been lacking in MSPRs from medical students who did not satisfy certain prerequisites; criteria not included in the assessed sample may have been present in other candidates’ documents. For example, if a student did not require remediation, details regarding original performance, type of remediation, and success of completion may not have been included. Finally, in selecting the criteria that were ultimately used for the data extraction, bias may have been introduced according to what the authors deemed to be important; others may value these criteria differently and may have included others as well.

No association was found between the medical schools’ criteria adherence and their geographic distribution, graduating class size, or age since establishment. Additionally, it is unclear as to why certain criteria were not included by any of the schools. Comments by the dean (or other senior official) may not have been included due to them being unfamiliar with the candidates on a personal level. As for standardized examination scores, their absence may be reflective of the greater trend towards devaluing/concealing objective performance data. These may represent areas for further investigation in the future.

The strength of this study lies in offering a comprehensive, pan-Canadian review of the current practice of medical school performance reporting. As it stands now, there is a risk that students from certain universities could unfortunately be disadvantaged in the application process, with their MSPRs and transcripts failing to detail their achievements to the same extent as their peers. Standardization may be necessary in order to facilitate fair comparison of graduates from different institutions. Implementation of objective data may allow more effective intra- and inter-scholastic comparison.

## Conclusions

Considerable heterogeneity exists in the information provided in official medical school documentation, as well as markedly little objective data. Standardization may be necessary in order to facilitate fair comparison of graduates from different institutions. Implementation of objective data may allow more effective intra- and inter-scholastic comparison.
